# Health-Promoting Effects, Phytochemical Constituents and Molecular Genetic Profile of the Purple Carrot ‘Purple Sun’ (*Daucus carota* L.)

**DOI:** 10.3390/nu16152505

**Published:** 2024-08-01

**Authors:** Viviana Maresca, Lucia Capasso, Daniela Rigano, Mariano Stornaiuolo, Carmina Sirignano, Sonia Piacente, Antonietta Cerulli, Nadia Marallo, Adriana Basile, Angela Nebbioso, Deborah Giordano, Angelo Facchiano, Luigi De Masi, Paola Bontempo

**Affiliations:** 1 Department of Biology, University of Naples Federico II, Via Cinthia 26, 80126 Naples, Italy; viviana.maresca@unina.it (V.M.); adriana.basile@unina.it (A.B.); 2 Department of Precision Medicine, University of Campania Luigi Vanvitelli, Via L. De Crecchio 7, 80138 Naples, Italy; lucia.capasso2@unicampania.it (L.C.); angela.nebbioso@unicampania.it (A.N.); paola.bontempo@unicampania.it (P.B.); 3 Department of Pharmacy, University of Naples Federico II, Via Montesano 49, 80131 Naples, Italy; mariano.stornaiuolo@unina.it (M.S.); carmina.sirignano@unina.it (C.S.); 4 Department of Pharmacy, University of Salerno, via Giovanni Paolo II 132, 84084 Fisciano (Salerno), Italy; piacente@unisa.it (S.P.); acerulli@unisa.it (A.C.); 5 Agronomist Consultant, Via S. Moccia 2/B, 83100 Avellino, Italy; nadia.marallo@libero.it; 6 Institute of Food Science (ISA), National Research Council (CNR), Via Roma 64, 83100 Avellino, Italy; deborah.giordano@isa.cnr.it (D.G.); angelo.facchiano@isa.cnr.it (A.F.); 7 Institute of Biosciences and BioResources (IBBR), National Research Council (CNR), Via Università 133, 80055 Portici (Naples), Italy

**Keywords:** *Daucus carota*, purple carrot variety, anthocyanins, antiproliferative effect, antioxidant and antibacterial activities

## Abstract

The purple carrot cultivar ‘Purple Sun’ (*Daucus carota* L.) is characterized by a relevant content of phenolic compounds and anthocyanins, which may play an important role in reducing the risk of chronic diseases and in the treatment of metabolic syndrome. In the present study, the genetic diversity, phytochemical composition, and bioactivities of this outstanding variety were studied for the first time. Genetic analysis by molecular markers estimated the level of genetic purity of this carrot cultivar, whose purple-pigmented roots were used for obtaining the purple carrot ethanol extract (PCE). With the aim to identify specialized metabolites potentially responsible for the bioactivities, the analysis of the metabolite profile of PCE by LC-ESI/LTQ Orbitrap/MS/MS was carried out. LC-ESI/HRMS analysis allowed the assignment of twenty-eight compounds, putatively identified as isocitric acid (**1**), phenolic acid derivatives (**2** and **6**), hydroxycinnamic acid derivatives (**9**, **10**, **12**–**14**, **16**, **17**, **19**, **22**, and **23**), anthocyanins (**3**–**5**, **7**, **8**, **11**, and **18**), flavanonols (**15** and **21**), flavonols (**20** and **24**), oxylipins (**25**, **26**, and **28**), and the sesquiterpene 11-acetyloxytorilolone (**27**); compound **26**, corresponding to the primary metabolite trihydroxyoctanoic acid (TriHOME), was the most abundant compound in the LC-ESI/HRMS analysis of the PCE, and hydroxycinnamic acid derivatives followed by anthocyanins were the two most represented groups. The antioxidant activity of PCE, expressed in terms of reactive oxygen species (ROS) level and antioxidant enzymes activity, and its pro-metabolic effect were evaluated. Moreover, the antibacterial activity on Gram (−) and (+) bacterial strains was investigated. An increase in the activity of antioxidant enzymes (SOD, CAT, and GPx), reaching a maximum at 0.5 mg/mL of PCE with a plateau at higher PCE concentrations (1.25, 2.5, and 5.0 mg/mL), was observed. PCE induced an initial decrease in ROS levels at 0.1 and 0.25 mg/mL concentrations, reaching the ROS levels of control at 0.5 mg/mL of PCE with a plateau at higher PCE concentrations (1.25, 2.5, and 5.0 mg/mL). Moreover, significant antioxidant and pro-metabolic effects of PCE on myoblasts were shown by a reduction in ROS content and an increase in ATP production linked to the promotion of mitochondrial respiration. Finally, the bacteriostatic activity of PCE was shown on the different bacterial strains tested, while the bactericidal action of PCE was exclusively observed against the Gram (+) *Staphylococcus aureus*. The bioactivities of PCE were also investigated from cellular and molecular points of view in colon and hematological cancer cells. The results showed that PCE induces proliferative arrest and modulates the expression of important cell-cycle regulators. For all these health-promoting effects, also supported by initial computational predictions, ‘Purple Sun’ is a promising functional food and an optimal candidate for pharmaceutical and/or nutraceutical preparations.

## 1. Introduction

Many epidemiological studies have well established that unhealthy diets and incorrect eating habits represent important risk factors for contracting non-communicable diseases [1,2], while eating foods rich in dietary flavonoids can contribute positively to our health [3,4]. Within these phytochemicals, the pigments anthocyanins (also known as anthocyans) are water-soluble phenolic compounds due to the presence of the flavylium (2-phenylchromenylium) ion in their base skeleton [5]. Originally isolated from flower petals and located in the cell vacuoles, anthocyanins are glycosides of the aglycons anthocyanidins and display a range of different colors (from dark blue to red) [5] in accordance with the conditions of pH, light, and temperature. They appear red under low pH conditions and turn blue with high pH (halochromism). Acylated forms show greater chemical stability and antioxidant activity than non-acylated ones [5]. Previous research has linked a high intake of anthocyanin-rich foods to a wide variety of health benefits, leading to increased longevity (including cancer, diabetes, and cardiovascular disease prevention) due, at least in part, to their antioxidant and anti-inflammatory effects [4,5,6]. Anthocyanins extracted from fruits and vegetables showed antiproliferative activity against multiple tumor cell types in vitro [7,8,9,10].

Cultivated carrot (*Daucus carota* L. ssp. *sativus* (Hoffm.) Schübl. and G. Martens, 1834), a diploid species (2n = 2x = 18) with a genome of 480 Mbp, is the most widely grown and consumed crop of the botanical family Apiaceae (Umbelliferae). Historical documents and recent genetic analysis dating back to about 1100 years ago indicate carrot domestication and its use as a storage root in areas of Central Asia (now Afghanistan), where the colors of the first carrot roots were yellow and purple [11]. It is highly likely that the widespread orange color of carrot root, due to high levels of alpha- and beta-carotene, is derived from domesticated yellow carrots [11]. To date, orange carrot is the most important source of provitamin A carotenoids worldwide, featuring anti-cancer and anti-aging effects [12,13]. Its production has constantly increased in recent years, being in the top ten vegetables for production area and market value (http://faostat.fao.org/faostat, accessed on 8 January 2024), thanks to, above all, the eating behavior of consumers who are more informed and aware of the health benefits associated with eating carrots. Carrot is mainly used in salads, soups, and juices, for colorant production in the food industry, and, more in general, for food preparation due to its high nutritional value [12,13,14,15]. The root of carrot is rich in crude fiber (cellulose, hemicellulose, and lignin), minerals (Ca, P, K, Fe, and Mg), appreciable amounts of vitamins (thiamin, riboflavin, niacin, folic acid, and ascorbic acid), amino acids, carbohydrates (sucrose, glucose, xylose, and fructose), phenols, and anthocyanins in the black/purple varieties [15,16].

In the present study, the carrot hybrid variety ‘Purple Sun’, characterized by deep purple roots (both outside and inside) and a sweet flavor, was investigated as a potential functional food and an important source of nutraceuticals. It was established that the purple color is due to high quantities of anthocyanins, almost exclusively cyanidin derivatives, which visually hide the orange of carotenes [12,14,16]. The variability of the natural compounds in the carrot roots is closely related to the genotype and to the degree of varietal genetic purity of the population, without considering environmental and cultivation conditions [17]. Consequently, the potential health and bioactivity effects of the purple root extract strongly depend on its genetic identity [17]. However, high varietal purity is difficult to maintain during production by the seed producer companies, allowing for a certain degree of genetic variability, so ascertaining the varietal genetic identity is essential. In this regard, molecular marker technologies are well known and reliable tools for genetic diagnostics [18]. To assess the genetic purity level of the purple carrot population under study, plants were subjected to DNA analysis. In this study, Random Amplified Polymorphic DNA (RAPD) markers were utilized because they have emerged among the most suitable markers for promptly assessing plant variety/hybrid purity due to their genome-wide presence and multi-locus nature [19,20].

To analyze the potential health effects and bioactivity of the carrot ‘Purple Sun’ pigmented root, we prepared an ethanol extract (PCE, Purple Carrot Extract) that was analyzed through liquid chromatography coupled to electrospray ionization with multiple-stage linear ion-trap and orbitrap high-resolution mass spectrometry (LC-ESI/LTQOrbitrap/MS/MS). LC-ESI/HRMS highlighted the occurrence of flavonoids, mainly anthocyanins, along with phenolic acid derivatives, hydroxycinnamic acid derivatives, isocitric acid, oxylipins, and a sesquiterpene.

Therefore, to highlight the potential nutraceutical value of the ‘Purple Sun’ carrot root, the antioxidant and pro-metabolic effect of PCE was evaluated on cultured myoblast, while the cellular and molecular mechanisms of PCE-induced antiproliferative effects were determined in colon (HCT116) and hematological (U937) cancer cells. In addition, antioxidant activity in terms of reactive oxygen species (ROS) levels and antioxidant enzyme activities in the polymorphonuclear leukocytes (PMNs) of healthy patients was evaluated, considering that ROS production has been demonstrated to contribute to the development of inflammatory diseases [21]. Finally, the antibacterial activity on nine bacterial strains, both Gram (−) and (+), was investigated.

## 2. Materials and Methods

### 2.1. Plant Material

The relatively recent hybrid commercial variety of purple carrot ‘Purple Sun’ (*Daucus carota* L.), developed in the Netherlands by Bejo Zaden BV, produces an intensely and uniformly colored deep purple root from the skin to the core with a sweet flavor, keeping its color once cooked [14]. In this study, ‘Purple Sun’ was cultivated during 2021 in open ground at the farm “Braccia Gerardo Carmine”, Morra De Sanctis (AV), High Irpinia, Italy. Purple carrot seeds were sown in spring, and carrot leaves and roots were harvested in summer.

### 2.2. Molecular Genetic Analysis of Purple Carrot Plants

The total genomic DNA of 10 purple carrot plants was isolated from 10 mg of leaves lyophilized by a freeze dryer (Alpha 1–2 LD plus, Martin Christ, Osterode am Harz, Germany) equipped with a vacuum pump (RZ 6, Vacuubrand, Wertheim am Main, Germany). Plant tissue disruption was achieved at dry-ice temperature through stainless steel beads in 2 mL Eppendorf tubes using a TissueLyser apparatus (Qiagen S.r.l., Milano, Italy) twice for 1 min at 30 Hz. DNA was extracted by using the GeneJET Plant Genomic DNA Purification Mini Kit (Thermo Fisher Scientific, Waltham, MA, USA) according to the manufacturer’s indications. After elution, the purity and quality of the DNA were checked on 2 μL samples by the 260/280 nm and 260/230 nm absorbance ratios in a UV-Vis Spectrophotometer (NanoDrop ND-1000, Thermo Fisher Scientific). DNA concentration was determined by absorbance at 260 nm. DNA integrity was assessed by agarose (1.5% *w*/*v*) gel electrophoresis. Finally, the DNA was diluted to operative concentrations of 1 ng/μL and stored at −20 °C.

The molecular genetic analysis of 10 purple carrot plants was performed by RAPD markers [19]. The arbitrary primers tested in the PCR reaction had 60–70% G + C content and were 10 nucleotides long (sequence details in Table S1). Each PCR was carried out in a final volume of 50 μL, containing 1× DreamTaq buffer (Thermo Fisher Scientific) with 2 mM MgCl_2_, brought to 3 mM MgCl_2_, 100 μM of each dNTP, 20 pmols of the unique arbitrary primer (Table S1), 2.0 Units of DreamTaq DNA polymerase (Thermo Fisher Scientific), and 10 ng of purple carrot genomic DNA. PCR amplifications were performed on a Veriti 96-Well Thermal Cycler with a heated lid (Applied Biosystems, Foster City, CA, USA) with the following cycling profile: initial DNA template melting for 3 min at 95 °C followed by 40 cycles each of DNA denaturation for 1 min at 95 °C, primer annealing for 1 min at 40 °C, and DNA synthesis for 1 min at 72 °C. The cycling ended with a final step at 72 °C for 10 min. Each reaction was repeated two times for each primer along with negative controls without genomic DNA. An aliquot of 25 μL reaction products was submitted to electrophoresis on 2% (*w*/*v*) agarose gel containing 0.5 μg/mL SyBr Safe and 1× TAE buffer (89 mM Tris-acetate at pH 8.4, 2 mM EDTA) at 5 V cm^−1^ for about 1.5 h. Amplicons were visualized by UV transillumination and digitalized by Gel Doc XR+ Gel Documentation System (Bio-Rad Laboratories S.r.l., Hercules, CA, USA).

After electrophoresis, the RAPD alleles corresponding to reproducible bands for each primer were scored as the number of bands per genotype, with “1” for the presence and “0” for the absence of each amplicon, based on the dominant genetic nature of RAPD markers. The binary data were used to calculate the genetic distance between pairwise genotypes according to Dice’s coefficient (Dc): 2Nij/(2Nij + Ni + Nj), where Nij is the number of bands common to the individuals i and j and Ni and Nj are the number of bands unique to the individuals i and j, respectively [22]. Therefore, Dc ranges from 0 to 1 and represents the fraction of RAPD alleles shared between two individuals, conferring twice the weight to the common bands with complete genetic identity corresponding to 1. The genetic relationships among the individual plants of purple carrot were estimated by the Dc triangular matrix and represented by a tree-like genetic diagram (dendrogram) constructed using the software PAST Ver. 4.13 [23], developed by Hammer et al. [24], according to the unweighted pair group method with the arithmetic mean (UPGMA) clustering algorithm [25]. To determine the relevant clusters, a cut-off line was depicted through the UPGMA dendrogram at the Dc mean value.

### 2.3. Preparation of Purple Carrot Extract (PCE)

To obtain PCE from the purple roots of the carrot ’Purple Sun’, the purple-pigmented roots of the 10 carrot plants previously analyzed by molecular markers were washed with running tap water, cut into slices of about 2–3 cm, and separately placed in a freeze dryer. Then, to constitute a single sample of 100 g representative of the 10 plants, 10 g per each carrot root were combined. After grinding root tissue with a pestle and mortar in the presence of liquid nitrogen, a homogenized aliquot of 100 mg was placed in a 500 mL flask containing 300 mL of aqueous ethanol (70%) and HCl (0.1%, *v*/*v*) and extracted for 30 min at 35 °C in a bath of sonicator water (20 kHz, Sonic, Rho Italy). Subsequently, the supernatant was filtered out. Hydrochloric acid has been added to prevent the degradation of non-acylated compounds [26]. The sonication extraction process was repeated three times in total. The ethanol extract obtained was centrifuged for 15 min in a centrifuge (3500× *g*) at room temperature, and then the ethanol was evaporated using a rotary evaporator (Heidolph laborota 4000 efficient, Heidolph Instruments GmbH & Co. KG, Schwabach, Germany) at a temperature not exceeding 40 °C. For the bioactivity assays, ethanol extract was resuspended in dimethyl sulfoxide (DMSO) and used at the final concentrations indicated in each experiment. DMSO alone was used as a negative control. Based on our previous studies, in which the bioactivities of total extracts were evaluated [7,9], we have found that the most effective biological doses are generally comprised in the range of concentrations from 0.1 to 5 mg/mL. Among these values, to test the antiproliferative activity of PCE, we performed a toxicity curve by MTT assay using 2.5 and 5 mg/mL concentrations, for which optimal results have been obtained.

### 2.4. LC-ESI/LTQOrbitrap/MS/MS Analysis

An LC-ESI/LTQOrbitrap/MS system was used for qualitative analysis using liquid chromatography coupled to electrospray ionization and high-resolution mass spectrometry consisting of a quaternary Accela 600 pump and an Accela autosampler coupled to a LTQOrbitrap XL mass spectrometer (ThermoScientific, San Jose, CA, USA), operating in negative electrospray ionization mode [27]. A Luna C_18_(2) 5 µm (150 mm × 2.00 mm) reversed-phase (RP) column (Phenomenex, Milano, Italy) was used for chromatographic separation with a flow rate of 0.2 mL/min. A binary system was used for the mobile phase, in particular, water (A) and acetonitrile (B); in both phases, 0.1% of formic acid was added. A linear gradient from 5 to 95% B in 30 min, held at 95% B for 5 min, was used, and 8 μL of PCE extract (0.5 mg/mL) were injected.

Specific parameters were set for the ESI source: sheath gas at 20 (arbitrary units), auxiliary gas at 5 (arbitrary units), source voltage at 3.5 kV, capillary temperature at 280 °C, capillary voltage at −48 V, and tube lens at −176.47 V. The mass range was from 150 to 1600 *m*/*z* with a resolution of 30,000. For the data-dependent scan, the first and the second most intense ions from the HRMS scan event were selected, with the aim to obtain tandem mass product ions with a normalization collision energy at 30% [28].

### 2.5. Reactive Oxygen Species (ROS) and Antioxidant Enzymes in Polymorphonuclear Leukocytes after Opsonized Zymosan Stress (OZ-Stressed PMNs)

PMNs were stressed with OZ to induce oxidative stress; then, the biological activities of PCE were evaluated on OZ-stressed PMNs. Whole blood was obtained with informed consent from healthy volunteers. Healthy fasting donors were subjected to peripheral blood sampling with K_3_EDTA vacutainers (Becton Dickinson, Plymouth, UK). PMNs were isolated following the protocol described by Badalamenti et al. (2021) [29]. Then, the isolated PMNs were treated with various PCE concentrations in the presence or absence of OZ.

The dichlorofluorescein (DCF) assay was carried out to quantify ROS by using the protocol of Manna et al. (2012) [30]. The PMNs were treated with PCE at concentrations of 0.1, 0.25, 0.50, 1.25, 2.5 and 5 mg/mL without or with OZ (0.5 mg/mL^−1^) following the protocol of Napolitano et al. (2022) [31]. The enzymatic activity of superoxide dismutase (SOD), catalase (CAT), and glutathione peroxidase (GPx) was determined in PMNs using commercial kit protocols (EnzyChrom, BioAssay System, San Diego, CA, USA) and expressed as U/L [32]. The experiments were performed in the presence and absence of OZ (0.5 mg mL^−1^) using PCE at concentrations of 0.1, 0.25, 0.50, 1.25, 2.5 and 5 mg/mL.

### 2.6. Antimicrobial Activity Assays: Minimum Inhibitory Concentration (MIC) and Minimum Bactericidal Concentration (MBC) Determination

Nine bacterial strains from the American Type Culture Collection (ATCC; Rockville, MD, USA) were employed. They included the Gram-positive (G+) bacteria *Staphylococcus aureus* (ATCC 13709) and *Enterococcus faecalis* (ATCC 14428) and the Gram-negative (G−) bacteria *Proteus mirabilis* (ATCC 7002), *Proteus vulgaris* (ATCC 12454), *Pseudomonas aeruginosa* (ATCC 27853), *Salmonella typhi* (ATCC 19430), *Enterobacter aerogenes* (ATCC 13048), *Enterobacter cloacae* (ATCC10699), and *Klebsiella pneumoniae* (ATCC 27736). 

The PCE was added to a 5 × 10^−2^ M DMSO stock solution and diluted from 0.01 to 1000 µg/mL serial concentrations in sterile physiological Tris buffer (pH 7.4, 0.05 M) immediately before being used [33].

Bacterial strains were grown on MH (Mueller Hinton) agar plates (DIFCO, Detroit, MI, USA) and suspended in MH broth (DIFCO). The MIC values against bacterial strains were determined using the broth-dilution method (MH broth) reported by Ericcson and Sherris (1971) [34]. The inoculum suspensions were prepared from 6 h broth cultures and adjusted to reach an OD_600_ of 1 unit. The extract was sterilized by filtration through Millipore filters (0.45 µm) and added to an MH broth medium. Serial 10-fold dilutions were made for a concentration range between 0.01 and 1000 µg/mL. Two-fold dilutions in the range between the minimum active and the maximum inactive concentrations were tested to obtain a more precise measure of the MIC. The bacterial suspensions were aerobically incubated for 24 h at 37 °C. The MIC was defined as the lowest concentration able to inhibit any visible bacterial growth. Cultures containing only a sterile physiological Tris buffer (pH 7.4, 0.05 M), which did not influence bacterial growth, were used as controls. The MIC values were also determined for tetracycline hydrochloride (Pharmacia, Milano, Italy), benzylpenicillin sodium (Cynamid, Catania, Italy), and cefotaxime sodium (Roussel Pharma, Milano, Italy) in MH broth using a standard method.

The MBC determination was carried out by transferring to the fresh MH broth aliquots of bacterial suspensions from the test tubes containing extract concentrations equal to or higher (up to 1000 µg/mL) than the MIC. The extract was tested in triplicate; the experiment was performed four times.

### 2.7. Cell Lines, Culture Conditions

C2C12 murine myoblasts, as a model of skeletal muscle, were cultured in Dulbecco’s modified Eagle Medium (DMEM) (Gibco, Grand Island, NY, USA), supplemented with 10% Fetal Bovine Serum (FBS) (Gibco), 2 mM Glutamine, 100 U/mL Penicillin, and 100 μg/mL Streptomycin (Gibco) at 37 °C in 5% CO_2_.

Acute myeloid leukemia (AML) U937 and human colon carcinoma cells HCT116 have been obtained from the ATCC. U937 cells were cultured in RPMI 1640 (Euroclone, Pero (MI), Italy), while HCT116 was cultured in Dulbecco’s Modified Eagle Medium (DMEM) with 10% Fetal Bovine Serum (Sigma–Aldrich, Milano, Italy), 2 mmol/L L-glutamine (Euroclone) and antibiotics (100 μg/mL penicillin, 100 μg/mL streptomycin, and 250 ng/mL amphotericin-B). Cells were grown at 37 °C with 5% CO_2_ and treated with PCE for 24 h and 48 h at the indicated concentrations [7].

### 2.8. Cell Cycle Analysis

Fluorescence-Activated Cell Sorting (FACS) analysis using a FACS Celesta Flow Cytometer (BD Biosciences, Milano, Italy) was used to determine the percentages of cell population in the different cell cycle stages and the percentage of cell death. After the PCE treatment, cells were collected and washed with phosphate-bufferedsaline (1× PBS). Then, cells were treated with a cycle buffer (1× PBS, 10% NP-40, 10% sodium citrate, and 2 mg/mL propidium iodide—PI) for 15 min at room temperature. To assess the percentage of the dead cell population, the cells were treated with PI buffer (1× PBS and 2 mg/mL PI) [7].

### 2.9. Total Protein Extraction and Western Blot Analysis

U937 and HCT116 cells were collected and washed three times with PBS 1× at 1200× *g* for 5 min at 4 °C, cell pellets suspended in lysis buffer (50 mmol/L Tris-HCl pH 7.4, 150 mmol/L NaCl, 1% NP40, 10 mmol/L NaF, 1 mmol/L PMSF, and protease inhibitor cocktail—PIC, Roche, Basel, Switzerland). The pellets were vortexed three times every 5 min and held at 4 °C. Finally, the samples were centrifuged at 13,000× *g* for 30 min at 4 °C, and the protein concentration was quantified by the Bradford assay (Bio-Rad) [9].

Thirty micrograms of the total protein extract were loaded onto 10% to 15% for sodium dodecyl sulfate-polyacrylamide gel electrophoresis (SDS-PAGE) and subsequently transferred to nitrocellulose membranes. The nitrocellulose filters were stained with Ponceau red (Sigma) as an additional control for an equal load. Then, the membranes were blocked with 5% milk, and Tris-Buffered Saline 1 M + 0.1% Tween (TBS-T) was used for membrane washings. The primary antibody was diluted in TBS-T and incubated at 4 °C overnight; then, the membranes were washed 3 times with TBS-T for 5 min followed by incubation with the horseradish peroxidase-conjugated (HRP) secondary antibodies diluted in milk 3% for Rabbit 1:10,000 and mouse 1:10,000 for 1 h at room temperature. Finally, immunoreactive proteins were visualized by enhanced chemiluminescence (ECL, Bio-Rad, Hercules, CA, USA) according to the manufacturer’s instructions with ChemiDoc XRS (Bio-Rad) and semi-quantified by densitometry using the Java-based image-processing and analysis software ImageJ 1.54a (U.S. National Institutes of Health, Bethesda, MD, USA).

The antibodies (Abs) used were c-Myc rabbit mAb (1:1000, 57–65 kDa, D84C12, Cell Signaling Technology, Danvers, MA, USA), anti-PARP1 rabbit pAb (1:1000, 130 kDa, ab137653, Abcam, Cambridge, UK), anti-Cyclin A2 rabbit pAb (1:1000, 54 kDa, ab137769, Abcam), anti-Cyclin D2 rabbit pAb (1:1000, 34 kDa, ab230883, Abcam), p16 INK4A Rabbit mAb (1:1000, 16 kDa, D7C1M, Cell Signaling Technology), and anti-Caspase-3 rabbit pAb (1:1000, 36 kDa,#9662, Cell Signaling). Hsp90 mouse mAb (1:1000, 90 kDa, ab13492, Abcam), GAPDH (1:3000, 37 kDa, E-AB-20059, Elabscience, Houston, TX, USA), and Anti-alpha tubulin rabbit pAb (1:1000, 54 kDa, ab4074, Abcam) were used to normalize the samples for equal loading. Secondary antibodies (CyDye™ 800; #GE29360790; #GE29360788; GE Healthcare-Amersham, Biosciences, Milano, Italy) were used with a dilution of 1:10,000. The chemiluminescent signals were detected using the ChemiDoc MP System (Bio-Rad).

### 2.10. ATP and Mitochondrial Stimulatory Activity of PCE in Muscle Cells

C2C12 cells were seeded into a 96-well plate at a density of 2 × 10^4^ cells/well in 100 μL of growth medium. Upon incubation with PCE for 72 h, intracellular ATP content assays were performed using an ATP assay kit (Toyo Ink, Tokyo, Japan), following the manufacturer’s instructions. At first, cells were washed twice with PBS. Next, 50 μL of serum-free DMEM and 50 μL of ATP assay reagent were added to each well. After 15 min of incubation, cells were washed twice with PBS to remove any residual media and unmetabolized substances. Subsequently, a lysis buffer from the ATP assay kit was added to the cells. The total cellular ATP content was measured by light emission with the Envision 2105 spectrofluorometer (Perkin Elmer, Waltham, MA, USA).

Mitochondria staining of primary myocytes was achieved by incubation with MitoTracker Red CMXRos (Thermo Fisher Scientific). A dye-working solution was prepared by diluting a stock solution (10 μM in DMSO) in DMEM to yield a final concentration of 100 nM. For staining of the in vitro samples, cells were rinsed twice in PBS before adding the dye. Thus, cells were incubated in the presence of the probe for 40 min in a cell incubator at 37 °C and 5% CO_2_. At the end of the incubation, cells and tissues were rinsed three times in DMEM and once in PBS, then fixed in 4% formaldehyde. Quantitative measurement of MitoTracker Red CMXRos fluorescence was performed with the spectrofluorometer Envision 2105.

### 2.11. Selection of Cyanidin Protein Targets and Molecular Docking

Cyanidin putative protein targets have been estimated searching by SwissTargetPrediction [35] on the basis of 2D and 3D structural similarities in the library of molecules with known activity on more than 3000 proteins. This system is able to screen which, and how likely, proteins are the intended targets for cyanidin, choosing as the cutoff for the results selection a probability value higher than 0.5. From the results obtained, only two proteins exceed the probability threshold: thrombin (UniprotID: P00734) and LXR-alpha (UniprotID: Q13133), with a probability value equal to 0.768 for both. For the docking simulation, the structure of cyanidin was obtained by the PubChem database [36] (CID: 128861) and converted in the .pdb format by Chimera 1.14 [37]. The structures of NAD-dependent protein deacetylase sirtuin-6 (SIRT6) in complex with cyanidin (used as control), of oxysterols liver X receptor (LXR)-alpha in complex with its agonist (GW-3965, PubChem CID: 447905), and thrombin in complex with sulfonamide inhibitor (PubChem CID 10095865) were retrieved from the PDB (6QCH, 3IPQ, and 2JH0, respectively) [38]. To evaluate the ability of cyanidin to bind effectively to these two protein targets, blind and focused docking simulations were performed by AutoDock 4.2.5.1 and AutoDockTools 1.5.6 [39]. In particular, for the focused docking, a 70 × 70 × 70 grid box was set with grid center coordinates equal to −24.959; 24.968; 21.184 for SIRT6; 41.415; 17.765; −5.323 for LXR-alpha; and 4.545; 19.034; and 47.581 for thrombin, while, for blind docking simulations, the grid dimension was set to 126 × 126 × 126 for all proteins. The default value was chosen for the spacing (0.375 Å). Results obtained have been analyzed by AutoDockTools and LigPlot v2.2 [40].

### 2.12. Statistical Analysis

ROS production, antioxidant activities, mitochondrial activity, and ATP content were examined by one-way analysis of variance (ANOVA), followed by Tukey’s multiple comparison post hoc test. In figures, values are presented as mean ± st. err; numbers not accompanied by the same letter are significantly different at *p* < 0.05. The cell cycle results from the FACS analysis are reported as the mean ± SD of three independent experiments.

## 3. Results and Discussion

### 3.1. Assessment of Genetic Diversity among Purple Carrot Plants

The quality and variability of the human diet strictly depends on the diversity of plants consumed, each possessing peculiar health properties. To properly determine the genetic purity and relationships among individual plants within the purple carrot population ‘Purple Sun’ in study, RAPD molecular markers were utilized [19]; i.e., DNA fragments obtained from PCR amplification of random regions of genomic DNA by using single primers of arbitrary nucleotide sequences. This procedure was chosen because RAPD has shown sensitivity in the identification and assessment of the genetic relatedness of plant clonal variants [18,41]. The RAPD-PCR procedure was consolidated in our previous works, showing sensitivity and reproducibility in the range of 1–100 ng genomic DNA [18,20,41].

In the present study, we used 10 ng of genomic DNA of purple carrot as a PCR template with 10 arbitrary decamer primers selected for reproducibility of the banding patterns (see Figure S1 and sequence details in Table S1). We also assayed arbitrary primers already utilized with success for the varietal discrimination of other species [18,20,41], surprisingly obtaining polymorphic markers (as in our previous works) despite the fact that carrot plants belong to the same variety (Table 1).

The RAPD profiles were effective in successfully identifying closely related varietal individuals. RAPD explored different regions of the genome, resulting in differences for each carrot individual of the same population in study (Table 1 and Figure S1). Alleles corresponding to reproducible amplicons, given the dominant genetic nature of RAPD markers, clearly identified a total of 111 markers or loci (Table 1) in 10 carrot individuals with 10 arbitrary primers for a total of 100 analyzed samples, resulting in 1110 data points (Figure S1). Out of these markers, 71 loci showed polymorphism (64%) that discriminated between carrot individuals and allowed us to assess their genetic relationships (Table S2). The numbers detected were dependent on the arbitrary primer used and on the single individual with an average of 11.1 loci per primer, going from a minimum of four bands for the primers AK10 and V06 to a maximum of 11 bands for the primers U1 and E14. Moreover, RAPD markers ranged from a minimum of 67 loci per CAR9 to a maximum of 83 loci per CAR8, with an average of 74.4 loci per sample. Surprisingly, more than one primer (AK10, E10, E11, and U1) yielded private markers, i.e., specific to a given individual and not shared with anyone else. These can be interesting for marker-assisted selection in breeding programs, where plants with private alleles can be exploited to produce genetic variability.

Genetic relationships are shown in Table S2, where the genetic similarities among all individual plants measured by Dc vary from 0.90 between CAR3 and CAR4 to 0.71 between CAR9 and CAR10. The total average Dc of 0.82 is indicative of the average genetic similarity, which corresponded to the complementary genetic distance value of 0.18. Then, the Dc genetic matrix (Table S2) was used to build the genetic tree (dendrogram) shown in Figure S2. Here, the average 0.82 Dc was used as a cut-off line to identify similarity clusters. The dendrogram topology grouped the carrot individuals into two main clusters constituted by six and three individuals, respectively, while only one individual was located apart (CAR1).

Molecular marker analysis suggested that each carrot individual of this population can boast its own genetic identity, even if belonging to the same variety ‘Purple Sun’. The presence of DNA variations allowed us to differentiate all the samples under study, even if they were extremely similar from a phenotypic point of view. Indeed, these polymorphisms highlight existing and inherited differences at the level of genomic DNA sequence, and they represent an optimal characterization system that can effectively integrate those based on the determination of phenotypic characteristics, often influenced by environmental conditions [17].

First research on the genetic diversity of carrots showed significant differences between wild and cultivated accessions [42]. Further studies revealed the history of carrot domestication [11,43]. More recent investigations have evidenced DNA differences among carrot varieties with orange, purple, and yellow roots. These results agree with the Afghan origin of carrot root with a purple color, genetically distant from carrot with orange-colored roots [44]. However, to our best knowledge, only few studies have used molecular markers also with the aim of showing the existence of genetic variability among individual carrot plants of the same variety [42,45]. According to the carrot breeding history, the present study revealed high levels of intra-cultivar heterogeneity in ‘Purple Sun’, apparently in contrast to the phenotypic stability.

In our study, RAPD analysis has proven to be a sensitive molecular tool for the genotyping of individual purple carrot plants and for determining the genetic purity level of carrot roots to produce crude extracts to utilize in subsequent applications, such as chemical compositional analysis and bioactivity assays. Finally, these findings can be particularly relevant in breeding programs and further and broader genetic purity studies of carrot cultivars and their derived products.

### 3.2. LC-ESI/LTQOrbitrap/MS/MS Analysis of Purple Carrot Extract

Taking into account the genetic results reported above, to define the phytochemical profile of PCE, an LC-ESI/LTQOrbitrap/MS/MS analysis was performed on a sample representative of 10 carrot plants, as reported in Section 2.3 of the Materials and Methods section. The LC-ESI/HRMS profile showed the occurrence of different classes of metabolites. The careful analysis of accurate mass, characteristic fragmentation pattern, retention time, as well as the literature data, allowed for the assignment of twenty-eight compounds (Table 2) putatively identified as isocitric acid (**1**), phenolic acid derivatives (**2** and **6**), hydroxycinnamic acid derivatives (**9**, **10**, **12**–**14**, **16**, **17**, **19, 22**, and **23**), anthocyanins (**3**–**5**, **7**, **8**, **11**, and **18**), flavanonols (**15** and **21**), flavonols (**20** and **24**), oxylipins (**25**, **26**, and **28**), and the sesquiterpene 11-acetyloxytorilolone (**27**).

**Figure 1:**
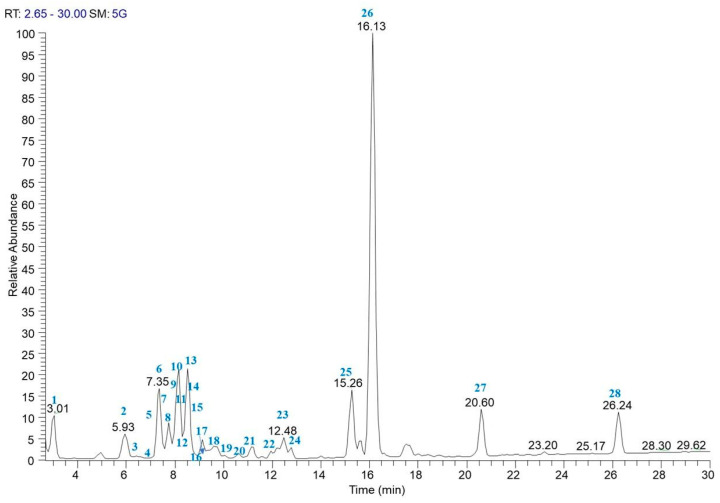
LC-ESI/LTQOrbitrap/MS profile (negative ion mode) of the purple carrot extract (PCE).

Anthocyanins and hydroxycinnamic acid derivatives were the two most represented groups of specialized metabolites in PCE extract (Figure 1) [46]. Anthocyanins were characterized by different aglycons with a sugar portion made up of two or three sugars linking, in some cases, sinapoyl, feruloyl, caffeoyl and *p*-coumaroyl moieties. In particular, compounds **3**, **4**, **5**, **7**, and **18** showed a characteristic fragment at *m*/*z* 285 corresponding to cyanidin, whereas compound **11** displayed a specific fragment at *m*/*z* 301 ascribable to peonidin; additionally, compound **8**, was characterized by a product ion at *m*/*z* 269 due to pelargonidin [47,48] (Figure 1, Table 2).

The accurate analysis of the LC-ESI/HRMS spectrum highlighted the occurrence of another two classes of flavonoids; in particular, peaks **20** and **24** were identified as the flavonol glycosides rutin and isorhamnetin-*O*-hexuronoside, respectively [49], whereas the careful analysis of the fragmentation pattern of compounds **15** and **21** allowed us to assign these peaks to dihydromyricetin (**15**) [50] and methoxydihydromyricetin (**21**), specialized metabolites belonging to the flavanonols class [51]. The second most representative class of specialized metabolites identified in the LC-ESI/LTQOrbitrap/MS/MS profile of PCE was represented by hydroxycinnamic acid derivatives (in detail, sinapoyl, caffeoyl, and feruloyl derivatives were detected). Herein, sinapoyl-*O*-hexoside (**9**) [52], feruloyl-*O*-di-hexosyl-pentoside (**12**), feruloyl-*O*-di-hexoside (**13**) [53], feruloyl-*O*-hexoside (**14**) [53], feruloyl-*O*-rutinoside (**16**) [54], diferuloyl-*O*-sucrose (**22**) [55], and diferuloyl-*O*-hexoside (**23**) [55], corresponding to the [M − H]^−^ pseudomolecular ions at *m*/*z* 385.1193, 649.1987, 517.1563, 355.1032, 501.1609, 693.2035, and 531.1512, respectively, yielded very simple fragmentation patterns in which the base peak was produced by the neutral loss of the sugar moiety. In particular, the loss of the hexose unit (neutral loss of 180 Da) was observed for **9**, **14**, **23**, the loss of the rutinose unit (neutral loss of 308 Da) was observed for **16**, and of the loss of two hexose units (neutral loss of 324 Da) was observed for **13** (Figure 1, Table 2). The fragmentation pattern of compound **12** (*m*/*z* = 649.1987) showed an ion at *m*/*z* 499 due to the loss of a pentose unit (neutral loss of 150), at *m*/*z* 355 due to the loss of a pentose and a hexose unit (neutral loss of 132 + 162), and *m*/*z* 193 due to the loss of a further hexose unit (neutral loss of 162) and attributable to a feruloyl moiety, suggesting for compound **12** the structure of feruloyl-*O*-di-hexosyl-pentoside. The fragmentation pattern of compound **22** (*m*/*z* = 693.2035) showed a product ion at *m*/*z* 499 due the loss of a feruloyl moiety (neutral loss of 194) and a product ion at *m*/*z* 337 due to the further loss of a hexose unit (neutral loss of 162), allowing us to hypothesize a diferuloyl-*O*-sucrose [55] (Figure 1, Table 2).

Analysis of peaks **10**, **17**, **19**, and the relative fragmentation pattern in comparison with the literature data allowed us to assign these peaks as 5-caffeoylquinic acid (**10**) [48], ferulic acid (**17**) [48], and 5-*O*-feruloylquinic acid (**19**) [48].

Protocatechuic acid-4-*O*-glucoside (**2**) [56] and 4-hydroxybenzoic acid 4-*O*-glucoside (**6**) [57] with pseudomolecular ion at *m*/*z* 315.0722 and 299.0772, respectively, were also putatively identified.

Moreover, the accurate analysis of the LC-ESI/LTQOrbitrap/MS/MS fragmentation pattern of peaks **1** and **27** revealed isocitric acid (**1**) [58] and 11-acetyloxytorilolone (**27**) [59] (Figure 1, Table 2).

Finally, the full MS spectrum of PCE highlighted three peaks (**25**, **26**, and **28**) ascribable to polar fatty acids belonging to the oxylipin class; in detail, compound **25** (*m*/*z* = 327.2178) and **26** (*m*/*z* = 329.2335) showed the same product ion at *m*/*z* 229. This was formed by neutral loss of 98 Da (for compound **25**) and 100 Da (for compound **26**) from the corresponding molecular ion by rearrangement of the acyl chain end-part and breakdown of the C12–C13 bond, highlighting the presence of an additional double bond in the end-part of compound **25** in comparison with **26**; therefore, compound **25** was assigned as Trihydroxyoctadecadienoic acid (TriHODE) and compound **26** as Trihydroxyoctanoic acid (TriHOME) [50]. In the same way, compound **28** was assigned to hydroxyoctadecadienoic acid (HODE) [49] (Figure 1, Table 2).

To the best of our knowledge, compounds **9**, **12**, **15**, **16**, **21**-**26**, and **28** have been identified here for the first time in *Daucus carota*, whereas compounds **1** [58], **2** [57], **6** [57], and **27** [59] have been reported here for the first time in a purple variety of *D. carota*. The remaining compounds have been previously reported in purple carrot [47,48,60].

### 3.3. Biological Activities of Purple Carrot Extract (PCE) and Potential Beneficial Effects on Human Health

Following the PCE phytochemical characterization by LC-ESI/LTQOrbitrap/MS/MS analysis, the next step was the determination of the PCE bioactivity. Biological effects of a plant extract are the result of its composition and of the interactions of its specific compounds, with plant genotype and environmental conditions playing important roles on its purity and quality. The genetic and chemical compositional data obtained in this study allowed guaranteeing PCE before assaying in vitro its bioactivities.

#### 3.3.1. Characterization of Antioxidant Activity of PCE

**Figure 2:**
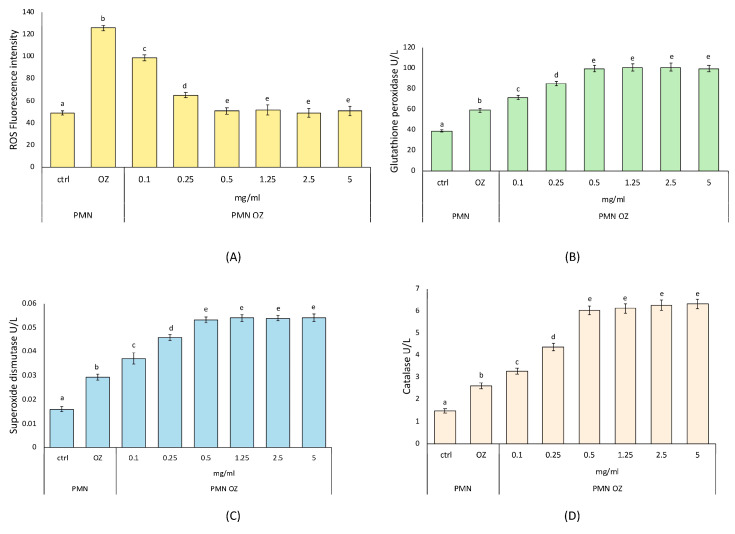
**Antioxidant activity of purple carrot extract (PCE).** ROS production (**A**) and activities of the anti-oxidant enzymes GPx (**B**), SOD (**C**), CAT (**D**) in opsonized zymosan (OZ)-stressed PMNs treated with PCE at the indicated concentrations. Data were presented as mean ± standard error. Numbers not accompanied by the same letter are significantly different at *p* value < 0.05.

The antioxidant activity was evaluated through ROS levels in polymorphonuclear leukocytes (PMNs) stressed with opsonized zymosan (OZ) to induce experimental sterile inflammation and then treated with PCE at different concentrations (see Section 2). As can be seen from Figure 2A, PCE causes a decrease in ROS levels in OZ-stressed PMNs starting already at low concentrations (0.1 and 0.25 mg/mL) but not reaching the ROS levels of untreated control PMNs. Then, in OZ-stressed PMNs treated with 0.5 mg/mL PCE, ROS levels become comparable to those observed in untreated control PMNs and remain constant with a plateau situation at greater PCE concentrations (1.25, 2.5, and 5.0 mg/mL).

As far as the activity of the antioxidant enzymes was concerned, SOD, CAT, and GPx show the same trend observed with the ROS levels. In OZ-stressed PMNs, PCE causes a gradual increase in the activity of these three enzymes, already at the lowest concentrations (0.1 and 0.25 mg/mL), compared to untreated PMNs. In more detail, for all three enzymes under study, the enzymatic activity reaches a maximum in OZ-stressed PMNs treated with 0.5 mg/mL PCE and then remains constant with a plateau situation at greater PCE concentrations (1.25, 2.5, and 5.0 mg/mL), as shown in Figure 2B–D.

Determining the ROS formation and activities of the main antioxidant enzymes, our results helped to elucidate how PCE influenced key aspects of cellular oxidative stress response [29,61]. In particular, we focused on the cellular model of PMNs, a type of white blood cell involved in inflammation and immune defense. Our findings showed that PCE has an interesting antioxidant activity, also suggesting the optimal PCE concentration of 5 mg/mL, as, at the same time, it reduced the production of free radicals and increased the enzymatic activity of antioxidant enzymes in treated PMNs compared to untreated control PMNs. This action also indicated a maximization effect of the cellular antioxidant defense systems. In fact, to tolerate mild oxidative stress, cells can contrast ROS production by increasing the expression levels of antioxidant enzymes, thereby restoring redox balance. Since ROS damage biological macromolecules, the antioxidant defense systems are responsible for cell survival. Once oxidative stress occurs, important biological molecules (proteins, nucleic acids, etc.) are damaged, leading to harmful consequences in terms of health. Since oxidative stress is involved in several pathologies, antioxidant enzymes play a key role as frontline defenders against free radicals [29,61]. In this context, the antioxidant effects of PCE can contribute to developing cellular adaptive mechanisms to contrast the oxidative stress and restore the redox balance.

**Figure 3:**
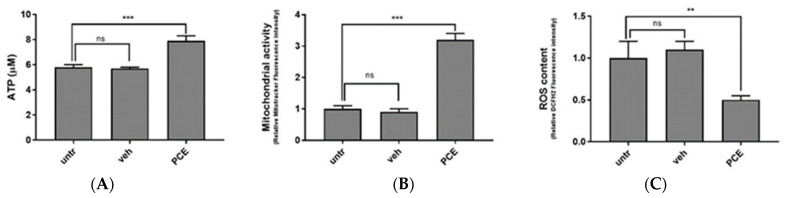
**Purple carrot extract (PCE) promotes ATP production, mitochondrial activity, and ROS reduction in cultured myoblasts.** Intracellular ATP content (**A**), mitochondrial activity (**B**) and ROS content (**C**) in C2C12 cells treated for 72 h in presence of PCE (300 μg/mL), an equal amount of vehicle (veh), or were left untreated (untr). Data are shown as mean ± SD of three independent experiments. Statistical analysis was performed by ANOVA test comparing each mean with that of untreated cells. *p* value = ** <0.01, *** <0.001, ns: non statistically significant.

Similarly to PMN cells, muscle cells are very sensitive to ROS, and several muscle conditions, including atrophy, muscle wasting, and sarcopenia, have been linked to increased muscular ROS content [62,63]. Together with altering muscle membrane lipids and proteins, oxidative stress affects muscle cell metabolism by reducing mitochondrial potential and hampering ATP production. In order to verify the effect of PCE on muscle cells, in vitro cultured C2C12 myoblasts were supplemented with 300 μg/mL of PCE for 72 h. Upon incubation, their ATP and ROS content, as well as their mitochondrial potential, were compared with untreated cells (untr) or with cells treated with the same amount of vehicle DMSO (veh). The ATP content was measured by luminescence assay. As shown in Figure 3A, and compared to untreated or vehicle-treated cells, 72 h of incubation with PCE promoted a statistically significant increase in ATP production (untr: 5.8 ± 0.2 μM; veh: 5.7 ± 0.1 μM; PCE: 7.9 ± 0.4 μM; *** = ANOVA *p* value < 0.001; n = 3 independent experiments).

To confirm that ATP stimulated by PCE was due to the promotion of mitochondrial respiration, we measured the intermembrane mitochondrial potential with the mitochondrial probe MitoTracker CMX-ROS. As shown in Figure 3B, myoblast cells treated with PCE showed increased mitochondrial activity compared to vehicle-treated cells (untr: 1.0 ± 0.1 fold increase in fluorescence; veh: 0.9 ± 0.1; PCE: 3.2 ± 0.2; *** = ANOVA *p* < 0.001; n = 3 independent experiments), confirming that PCE stimulates mitochondrial activity.

Finally, as expected, PCE also presents antioxidant activity in muscle cells. As shown in Figure 3C, DCFH2 staining of human myoblasts cells treated with PCE showed reduced ROS content when compared to cells treated with vehicle (untr: 1.0 ± 0.2 relative fluorescence (a.u.); veh: 1.1 ± 0.1; PCE: 0.5 ± 0.05; ** = ANOVA *p* value < 0.01; n = 3 independent experiments), confirming that, also in muscle cells, PCE contributed to maintaining endogenous ROS homeostasis.

#### 3.3.2. Determination of Antibacterial Activity of PCE

The antibacterial activity of PCE on nine bacterial strains, both Gram-negative (−) and Gram-positive (+) species, was investigated, showing that PCE has important effects against Gram (+) bacteria (*Staphylococcus aureus* and *Enterococcus faecalis*) and Gram (−) bacteria (*Proteus vulgaris*, *Proteus mirabilis*, *Salmonella typhi*, *Enterobacter cloacae*, *Enterobacter aerogenes*, *Pseudomonas aeruginosa*, and *Klebsiella pneumoniae*) (Table 3). The results of the MIC determination, i.e., the lowest concentration of PCE able to inhibit bacterial growth, highlighted the greater inhibitory efficacy of PCE against the Gram (+) *S. aureus* and *E. faecalis*, followed by intermediate inhibitory efficacy on the Gram (−) *P. mirabilis*, *P. vulgaris*, and *E. cloacae*, and lower efficacy against *S. typhi* and *E. aerogenes*, while *P. aeruginosa* and *K. pneumoniae* showed the least sensitivity to PCE (Table 3). The MIC values were also determined for comparison with the reference antibiotics cefotaxime sodium (CTAX), benzyl penicillin sodium (PENG), and tetracycline hydrochloride (TET) (Table 3). Moreover, the MBC determination was carried out by using PCE concentrations equal to or higher (up to 1000 µg/mL) than the MIC. The results showed the bactericidal action of PCE against the Gram (+) *S. aureus*, while no effect was detected on other bacterial strains (Table 3). All this considered, the antimicrobial action of PCE could contribute to restraining the bacteria growth mainly by an inhibitory mechanism, with efficacy closely depending on the bacterial strain considered, mainly against the Gram (+) *S. aureus*, especially considering that some bacteria were resistant to the reference antibiotics.

#### 3.3.3. Analysis of Antiproliferative Activity of PCE on Hematological and Epithelial Cancer Cells

**Figure 4:**
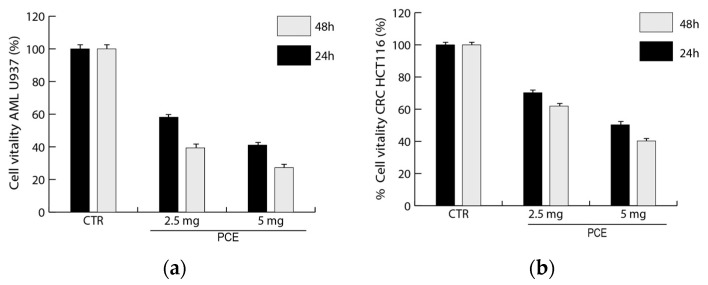
**Effects of purple carrot extract (PCE) on the vitality of tumor cells.** The inhibitory effects of different concentrations of PCE (0–2.5–5 mg/mL) on U937 (**a**) and HCT116 (**b**) cells were evaluated by MTT assay and expressed as a percentage of cell vitality compared to the untreated control (CTR). Values are mean ± standard deviation (SD) of biological triplicates.

The PCE antiproliferative effects were determined in colon (HCT116) and hematological (U937) cancer cells. Cell vitality after PCE treatment was evaluated in vitro by MTT assay on U937 and HCT116 cell lines. The analysis of U937 and HCT116 cells treated with PCE showed important bioactivity effects for both tested cell lines, also highlighting differences in the response to PCE (Figure 4). In leukemic cell line U937, a reduction in cell vitality from about 40% to 60%, going from 24 to 48 h, respectively, was already observed at 2.5 mg/mL of PCE. Then, a treatment with a higher concentration of PCE at 5 mg/mL was able to induce a reduction in U937 vitality from about 60% to 70%, going from 24 to 48 h, respectively. HCT116 cells appeared slightly less sensitive than U937 to the PCE treatment; in fact, we can observe a reduction in cell vitality from about 30% to 40%, going from 24 to 48 h, respectively, already at 2.5 mg/mL of PCE (Figure 4). Moreover, a treatment with PCE at 5 mg/mL was able to induce a reduction in HCT116 vitality from about 50% to 60%, going from 24 to 48 h, respectively. A time-dose-dependent reduction in vitality was observed for both cell lines, with a greater effect after 48 h at the maximum concentration used.

**Figure 5:**
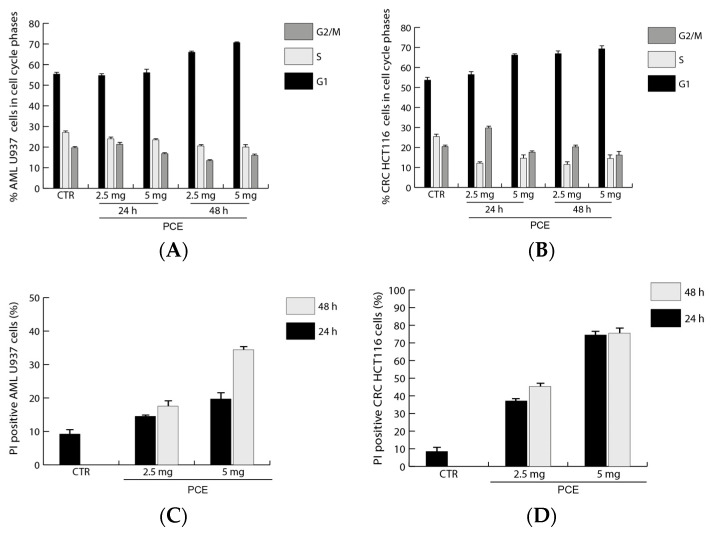
**Analysis of cell cycle progression after treatment with purple carrot extract (PCE).** Cell cycle (**A**,**B**) and cell death assays with propidium iodide (PI) staining (**C**,**D**) by FACS analysis, respectively. U937 (**A**,**C**) and HCT 116 (**B**,**D**) cells were treated with PCE at the indicated doses and times. Values are mean ± standard deviation (SD) of biological triplicates.

The data obtained after treatment with PCE from the cell vitality analysis conducted using the MTT assay are in line with the results from the cell cycle analysis of U937 and HCT116 carried out by flow cytometry. Findings showed differences in the effects of PCE treatment for the two tested cell lines related to their different sensitivity to the extract. In U937, the treatments with PCE at 2.5 mg/mL and 5 mg/mL were able to induce marked variations in cell cycle progression only after 48 h through an increase in G1 and a reduction in S and G2/M phases in comparison to the untreated control and to the treatment at 24 h (Figure 5A). Cell cycle analysis in HCT116 showed that the treatment with PCE at 2.5 mg/mL was able to induce marked variations in cell cycle progression already after 24 h, and until 48 h, through an increase in G1 and a reduction in the S and G2/M phases in comparison to the untreated control (Figure 5B). The treatment with PCE at both concentrations showed important variations in HCT116 cell number already after 24 h, increasing in G1 and reducing in the S and G2/M phases, while also persisting after 48 h (Figure 5B).

The percentage of dead cells after treatment with 2.5 and 5.0 mg/mL PCE was assessed by flow cytometry following the staining of cells with propidium iodide (PI). For U937 cells of acute myeloid leukemia, there was an increase in cell death compared to the control after just 24 h at a concentration of 2.5 mg/mL PCE (Figure 5C). This same effect increased with 5 mg/mL PCE after 24 h. The trend was similar after 48 h of PCE treatment, reaching the maximum of cell death (about 35%) with 5 mg/mL PCE, as shown in Figure 5C. In HCT116 cells of colon cancer, a trend similar to that seen in U937 is noted, but with a greater effect (Figure 5D). Cell death appears to be time-dose dependent in both cases. In fact, also for HCT116, the maximum effect is reached after 48 h of treatment at a concentration of 5 mg/mL PCE, reaching, in this case, the maximum of cell death (75%), as shown in Figure 5D.

Nowadays, the spectrum of beneficial effects of plant-based foods containing high levels of anthocyanins is clearly emerging [12,13,64,65,66], including the antiproliferative action. On this side, the analysis of protein expression by Western blot allowed us to investigate on the molecular level the antiproliferative effect induced by PCE, evaluating protein factors involved in the progression of the cell cycle. This analysis was performed on U937 cells, as they have shown an important reduction in cell vitality following PCE treatment. In detail, the molecular targets were the proteins Myc, p16, caspase-3, PARP-1 and cyclins A2 and D2, whose expression was evaluated after PCE treatment (24, 48 h) at two different concentrations (2.5, 5.0 mg/mL).

A strong repression of the Myc protein was observed after just 24 h of treatment with PCE at both concentrations (Figure S3). Myc proteins represent a family of transcription factors that are fundamental for the activation of genes involved in cell proliferation [67]. An altered regulation of the *Myc* gene can result in an overexpression of the protein itself and, consequently, in the loss of control of cell proliferation, typical of tumor cells. In fact, the *Myc* gene is dysregulated in a large number of tumors. The inhibition of its expression obtained through PCE treatment is therefore an important finding in support of PCE bioactivity.

Then, the PCE treatment induced an increase in the expression of p16, especially visible after 48 h, being time-dependent (Figure S3). P16 is a key factor in regulating the cell cycle [68]. In fact, its action takes place by binding cyclin D and preventing its interaction with cyclin-dependent kinase 4 (CDK4). So, the cyclin D-CDK4 complex can not be formed and the transition from the G1 phase to the S phase of the cell cycle does not occur. In this way, it prevents cells from proliferating uncontrollably and is, in fact, defined as a tumor suppressor protein. Thus, its increase would suggest that the PCE treatment could inhibit uncontrolled proliferation.

PCE also increased the cleavage of caspase-3, a proteolytic enzyme that, when activated, cuts key proteins to cellular life, leading to apoptosis [66]. This mechanism is fundamental for the removal of damaged cells and is finely regulated. However, an altered regulation can lead to various pathological conditions, including cancer. Its cleavage, which indicates its own activation, was accompanied by increased expression already after 24 h, with a maximum effect at the concentration of 5 mg/mL PCE (Figure S3). These variations indicated that the apoptotic pathway is involved in PCE antiproliferative activity. Indeed, PCE also induced the cleavage of PARP (Figure S3). During apoptosis, caspase-3 can cleave PARP, inactivating it and making it unable to repair DNA damage; this can lead to the phenomenon of apoptosis. PARP cleavage is therefore considered a marker of apoptosis [66].

PCE showed a dose-dependent inhibition of expression on both cyclins (A2 and D2); in fact, it showed a greater effect at the concentration of 5 mg/mL for both treatment times (Figure S3 for cyclin A2 and cyclin D2). Cyclin A2 plays a fundamental role in regulating the progression of the cell cycle, particularly in the S and G2 phases, contributing to DNA replication and preparing the cell for mitosis, while cyclin D2 promotes the progression from the G1 phase to the S phase. Therefore, an inhibition of their expression indicates a block in the progression of the cell cycle [68]. The results obtained indicate that PCE was able to down-regulate cyclins A2 and D2, with consequent inhibition of cell cycle progression.

At the same time, an increase in the cleavage of caspase-3 mediated PARP was noted, suggesting that the cells undergo apoptosis. Overall, although further studies are needed, these several effects indicate a significant impact of PCE on the mechanisms involved in the cell-cycle regulation, strongly suggesting potential antiproliferative properties.

Considering all the bioactivity results of the present study, the ‘Purple Sun’ carrot root could represent a promising functional food and an optimal candidate for pharmaceutical and/or nutraceutical preparations; additionally, it could also aid in preventing cancer, regulating blood sugar levels, balancing oxidation, and fighting aging.

#### 3.3.4. Prediction of Cyanidin Protein Targets and Molecular Docking

The abundance of cyanidin derivatives in the purple roots of ‘Purple Sun’ (Table 2) prompted us to verify the cyanidin protein targets of potential therapeutic interest based on the direct physical target–ligand interaction through in silico testing. After an in silico screening, during which proteins were predicted as potential targets for cyanidin, only LXR-alpha and thrombin exceeded the probability threshold and could be considered as possible candidates. Moreover, to confirm the cyanidin binding to these two proteins, blind and focused docking simulations were also performed.

To allow for a comparison of the binding energy and of the conformation adopted by cyanidin, redocking simulations have been performed for the complex SIRT6-cyanidin present in the PDB database, the only available complex between cyanidin and a protein. Focused docking simulations for this complex highlight a binding energy for the ligand that varies from −6.73 kcal/mol to −7.31 kcal/mol for the crystallized complex (Table S3), even if the blind simulations show the possibility for this compound to bind also to another area of the protein with a better energy (−8.39 kcal/mol). These values have been used as a reference for attributing a real significance to the results obtained with the protein targets suggested by this study. Cyanidin is known to be a potent SIRT6 activator, determining protection against metabolic and aging-related diseases [69].

Docking results obtained for LXR-alpha disclose, as expected, that the focused docking procedure is able to reproduce the crystallographic data in complex with its GW3965 synthetic agonist. However, in blind mode, docking simulations apparently suggest a preference for another binding site. GW3965 is highly affine for the receptor, with a binding energy equal to −14.38 kcal/mol. Cyanidin shows a high specificity for the agonist-binding zone (Figure S4A) that is detected both by blind and focused docking modes. Its binding energy is lower than −8.50 kcal/mol, which is even better by almost 1 kcal/mol than the one detected for the crystallographic complex Sirt6-cyanidin, or at least can be considered comparable to the best result obtained for the cyanidin interaction with the entire surface of this protein. GW3965 is a potent activator of LXR-alpha after its ingestion in mice. An increased activity of the reverse cholesterol transporter ABCA1 both in the small intestine and in peripheral macrophages is observed. Moreover, it induces an increasing in the plasma concentrations of HDL cholesterol [70]. The binding site of this agonist is well characterized by crystallography, with residues involved in H-bonds (Table S3) [71]. The binding of cyanidin to the same residues involved in the GW3965 binding and the formation of three H-bonds with three of them (Figure S4B,C) leads to the hypothesis that cyanidin may act as GW3965. Another possible role of cyanidin in the activation of LXR-alpha is suggested by a study on the cyanidin-3-*O*-β-glucoside (C3G). This compound promotes the LXR-alpha activation that, in turn, induces the efflux of cholesterol and 7-oxysterols from the human aortic endothelial cells by ATP-binding cassette transporter G1 mediation [72]. Therefore, cyanidin binding to the agonist GW3965 site on LXR-alpha suggests that it could have an important role in the prevention of atherogenesis and endothelial cell toxicity due to oxidized sterols.

Similar docking simulations have been performed between cyanidin and thrombin and between thrombin and its sulfonamide inhibitor (called SI for simplicity) [73]. The re-docking simulation highlights that SI binds the active site of thrombin, where the catalytic triad (H57-D102-S195) is located with a mean binding energy equal to almost −9.00 kcal/mol in the focused simulation. The blind simulation highlights the specificity of this inhibitor for the pocket detectable from the crystallographic complex, even if the conformation of the ligand appears to vary between two intermediate forms compared to the crystallized one, with the mean binding energy being slightly lower than the focused docking simulation (−8.76/−8.01 kcal/mol). Cyanidin is very selective for the active site pocket, with an interaction pose confirmed both from focused and blind simulations, as visible from the residues of interaction identical to SI (Table S3). Cyanidin seems to create stable contacts, forming four H-bonds, among which the one with the catalytic residue S195 and the other with G219 stand out, shared also by SI. The binding energy values obtained are in line with the control SIRT6–cyanidin complex, with the lowest binding energy being equal to approximately −7.70 kcal/mol and the mean being equal to −7.20 kcal/mol. Moreover, the binding pose of cyanidin is in part superimposable to the SI crystallographic pose (Figure S5), therefore suggesting a possible inhibition role of this molecule towards thrombin. Cyanidin experimentally showed a strong inhibition effect on thrombin, reducing in a dose-dependent manner the initial velocity of fibrin polymerization [74]. Moreover, other experimental studies have demonstrated that cyanidin inhibits the activity of coagulation factor X, necessary for thrombin activation [75]. In this view, cyanidin seems to prevent thrombin activity by acting on multiple levels.

These first computational results suggest how cyanidin has further potential therapeutic targets, even if not directly related to each other and to the cell cycle players, being able to achieve its beneficial effects through different paths of molecular interaction.

## 4. Conclusions and Perspectives

The results on the bioactivity of PCE in vitro are closely related to the specific pigmented carrot genotype, because the biological effects are a direct consequence of the influence of the peculiar phenolic pattern of PCE from this purple variety. However, in our study, the observed bioactivity cannot be attributed to a single molecule or class of compounds. Instead, it is much more likely that a synergistic effect of the different specialized metabolites on the bioactivity can exist. Therefore, further studies should be carried out for attributing the real mechanisms of action to the main compounds and to their combination. Moreover, it is known that the beneficial effects of a polyphenol and anthocyanin-rich diet are due to the complex mixture of specialized metabolites present in the plant extract [66]. However, the response to an in vivo treatment with PCE might be also influenced by the particular dietary regime. We could speculate that different diets might change the outcome of treatment. It is known that metabolism and the absorption of anthocyanins occur primarily in the gastrointestinal tract in the form of smaller bioavailable molecules derived from the parent anthocyanins, while their concentration in the bloodstream is very low as a consequence of minimal systemic bioavailability (0.26–1.80%) [7,9]. Therefore, the beneficial effects of an anthocyanin-rich diet are presumably related to the smaller metabolites that concentrate in the body at high doses for long time. It is very difficult to obtain precise information on the effective concentration of anthocyanins in body fluids and tissues, even if we know their exact concentration in the plant extract. The absorption and bioavailability of these compounds after oral administration play key roles in their potential use in disease prevention. However, further studies are required to demonstrate that/how the low in vivo concentrations of anthocyanins can be sufficient to elicit beneficial effects. Moreover, pharmacological research and clinical trials should verify the safety to be able to use higher concentrations of anthocyanins. All these aspects also point out that further investigation into the role of anthocyanin metabolites is expected.

Based on our results, the total extract of *D. carota* variety ‘Purple Sun’ has been shown to have an important biological potential for both antioxidant and antiproliferative activities, as guaranteed by genetic and chemical analyses. The treatment was able to reduce ROS levels with great effectiveness. The antiproliferative activity analyzed on two tumor cell lines showed equally promising results. In fact, the extract managed to block both cell lines in the G1 phase. This is also visible at a molecular level based on Western blots carried out on specific proteins responsible for controlling cell cycle progression. In particular, the treatment inhibits the expression of cyclins, indicating an arrest in the progression of the cell cycle. This effect is further accentuated by the increase in the expression of p16 protein, a known inhibitor of cyclin-dependent kinases. Finally, the increase in caspase 3 and PARP cleavage indicates that proliferative arrest may be related to the restoration of the apoptotic program. These experimentally demonstrated bioactivities, along with those in silico predicted, make the purple carrot ‘Purple Sun’ particularly interesting for its potential use in the treatment of tumors and other pathologies. The results of this study, alongside that of others carried out on natural extracts, indicate how plant-based bioactive compounds can be an important resource to consider for the development of new drugs and therapeutic strategies. In the near future, our results might also furnish a contribution to the reintroduction of purple carrot cultivation for a food market of higher quality.

## Figures and Tables

**Table 1:** DNA bands amplified by 10 RAPD arbitrary primers in 10 individual plants of purple carrot ‘Purple Sun’.

Primers	CAR1	CAR2	CAR3	CAR4	CAR5	CAR6	CAR7	CAR8	CAR9	CAR10	Polymorphic Loci/Total Loci	Private Loci
**AK10**	6	5	6	7	5	4	6	8	5	5	7/10 (70%)	1
**E10**	8	9	8	8	9	7	7	8	8	8	3/10 (30%)	1
**E11**	8	9	9	9	9	9	10	9	8	8	5/12 (42%)	2
**E14**	9	8	11	11	9	9	8	8	5	9	10/12 (83%)	0
**U1**	7	7	7	8	8	7	10	11	7	8	9/13 (69%)	1
**U3**	7	8	8	10	8	9	7	7	7	9	4/10 (40%)	0
**U4**	7	10	7	6	6	9	6	8	8	7	9/11 (82%)	0
**U19**	7	9	8	8	8	9	9	10	8	8	6/12 (50%)	0
**V01**	6	5	7	6	5	5	6	8	5	6	12/12 (100%)	0
**V06**	6	4	5	5	5	6	6	6	6	6	6/9 (67%)	0
**Private bands**	1	2	0	0	0	0	1	1	0	0		5/111 (4.5%)
**Total bands**	71	74	76	78	72	74	75	83	67	74	71/111 (64%)	

**Table 2:** Metabolites putatively identified in the purple carrot extract (PCE) by LC-ESI/LTQOrbitrap/MS/ analysis.

#	Compound	*R_t_* (min)	Molecular Formula	[M − H]^−^	[M − 2H]^−^	Error (ppm)	Characteristic Product Ions
**1**	isocitric acid **	3.01	C_6_H_8_O_7_	191.1200		4.5	147, 111
**2**	protocatechuic acid 4-*O*-glucoside **	5.93	C_13_H_16_O_9_	315.0722		3.53	153
**3**	cyanidin 3-*O*-xylosyl-sinapoylglucosyl-galactoside *	7.05	C_43_H_49_O_24_^+^		947.2468	1.68	785, 285
**4**	cyanidin 3-*O*-xylosyl-feruloylglucosyl-galactoside *	7.35	C_42_H_47_O_23_^+^		917.2361	1.67	285
**5**	cyanidin 3-caffeoylsophoroside 5-glucoside *	11.60	C_42_H_47_O_24_^+^		933.2008	1.33	285
**6**	4-hydroxybenzoic acid 4-*O*-glucoside **	7.50	C_13_H_16_O_8_	299.0772		3.66	137
**7**	cyanidin 3-*O*-xylosyl-p-coumaroylglucosyl-galactoside *	7.50	C_41_H_45_O_22_^+^		887.2259	2.14	527, 285
**8**	pelargonidin 3-*O*-xylosyl-feruloylglucosyl-galactoside *	8.86	C_42_H_47_O_22_^+^		901.2408	1.20	269
**9**	sinapoyl-*O*-hexoside	8.63	C_17_H_22_O_10_	385.1193		2.13	205
**10**	5-caffeoylquinic acid *	8.02	C_16_H_18_O_9_	353.0881		3.94	191, 135
**11**	peonidin 3-*O*-xylosyl-galactoside *	8.09	C_27_H_31_O_15_^+^		593.1665	1.27	301
**12**	feruloyl-*O*-di-hexosyl-pentoside	8.63	C_27_H_38_O_18_	649.1987		1.29	499, 397, 355, 193
**13**	feruloyl-*O*-di-hexoside *	8.55	C_22_H_30_O_14_	517.1563		2.16	337, 193
**14**	feruloyl-*O*-hexoside *	8.73	C_16_H_20_O_9_	355.1032		2.48	175
**15**	dihydromyricetin	8.88	C_15_H_12_O_8_	319.0457		2.75	285, 193
**16**	feruloyl-*O*-rutinoside	9.12	C_22_H_30_O_13_	501.1609		2.22	397, 193
**17**	ferulic acid *	9.12	C_10_H_10_O_4_	193.0504		4.38	178, 149
**18**	cyanidin-3-*O*-xylosyl-galactoside *	9.35	C_26_H_29_O_15_^+^		579.1358	3.73	417, 285
**19**	5-*O*-feruloylquinic acid *	9.63	C_17_H_20_O_9_	367.1035		2.97	193
**20**	Rutin *	10.05	C_27_H_30_O_16_	609.1456		1.02	463, 301
**21**	methoxy-dihydromyricetin	11.94	C_16_H_14_O_8_	333.0615		3.10	283, 185
**22**	diferuloyl-*O*-sucrose	12.17	C_32_H_38_O_17_	693.2035		1.39	517, 499, 337
**23**	diferuloyl-*O*-hexoside	12.48	C_26_H_28_O_12_	531.1512		2.67	351, 193
**24**	isorhamnetin-*O*-hexuronoside	12.79	C_23_H_24_O_12_	491.1193		1.75	459, 315, 151
**25**	trihydroxyoctadecadienoic acid (TriHODE)	15.26	C_18_H_32_O_5_	327.2178		3.66	291, 229, 211, 171
**26**	trihydroxyoctanoic acid (TriHOME)	16.13	C_18_H_34_O_5_	329.2335		3.85	293, 229, 211, 171
**27**	11-acetyloxytorilolone **	20.60	C_17_H_26_O_4_	293.1759		4.07	236, 221
**28**	hydroxyoctadecadienoic acid(HODE)	26.24	C_18_H_32_O_3_	295.2278		3.62	277, 195, 171

* Previously reported in purple *D. carota*. ** Previously reported in *D. carota*.

**Table 3:** MIC and MBC values (µg/mL) of purple carrot extract (PCE) with reference antibiotics (see notes).

	MIC					MBC
	ATCC	PCE	CTAX	PENG	TET	PCE
*Staphylococcus aureus*	13709	**3.9**	2	0.03	2	**62.5**
*Enterococcus faecalis*	14428	**3.9**	R	8	2	**R**
*Proteus vulgaris*	12454	**15.6**	2	4	R	**R**
*Proteus mirabilis*	7002	**15.6**	0.03	4	32	**R**
*Salmonella typhi*	19430	**31.3**	0.5	4	1	**R**
*Enterobacter cloacae*	10699	**15.6**	R	4	R	**R**
*Enterobacter aerogenes*	13048	**31.3**	R	4	R	**R**
*Pseudomonas aeruginosa*	27853	**62.5**	16	R	32	**R**
*Klebsiella pneumoniae*	27736	**62.5**	0.1	R	16	**R**

PCE in bold, CTAX = cefotaxime, PENG = benzyl penicillin sodium, TET = Tetracycline, R = resistant.

## Data Availability

Data is contained within the article and Supplementary Materials.

## Supplementary Materials

The following supporting information can be downloaded at: https://www.mdpi.com/article/10.3390/nu16152505/s1. Figure S1. Genetic diversity of purple carrot ‘Purple Sun’ detected by RAPD molecular analysis. Figure S2. Dendrogram based on UPGMA clustering of RAPD markers from purple carrot ‘Purple Sun’ plants. Figure S3. Purple carrot extract (PCE) modulated the expression of important cell cycle players in hematological cancer cells. Figure S4. Interactions of cyanidin and GW3965 in the agonist pocket of LXR-alpha. Figure S5. Interactions of cyanidin and SI in the active site pocket of thrombin. Table S1. Details of the 10-mer arbitrary primers used for DNA analysis of the purple carrot ‘Purple Sun’ in study. Table S2. Similarity/distance matrix based on Dice coefficient between pairs of individual samples in study belonging to the purple carrot ‘Purple Sun’. Table S3. Results of the molecular docking simulations.
