# Supplementary material for: Health-Promoting Effects, Phytochemical Constituents and Molecular Genetic Profile of the Purple Carrot ‘Purple Sun’ (Daucus carota L.)
Source: Nutrients. 2024 Aug 1;16(15):2505. doi: 10.3390/nu16152505 (PMC11314219; doi:10.3390/nu16152505)
Supplement: Supplementary file 1 [file nutrients-16-02505-s001.zip › nutrients-3101593-supplementary.pdf]

SUPPLEMENTARY MATERIAL

Health Promoting Effects, Phytochemical Constituents and Molecular Genetic Profile of the Purple Carrot 'Purple Sun' (*Daucus carota* L.)

Viviana Maresca [1,†], Lucia Capasso [2,†], Daniela Rigano [3,*], Mariano Stornaiuolo [3], Carmina Sirignano [3], Sonia Piacente [4], Antonietta Cerulli [4], Nadia Marallo [5], Adriana Basile [1], Angela Nebbioso [2], Deborah Giordano [6], Angelo Facchiano [6], Luigi De Masi [7,*] and Paola Bontempo [2]

[1] Department of Biology, University of Naples Federico II, Via Cinthia 26, 80126 Naples, Italy; viviana.maresca@unina.it (V.M.); adriana.basile@unina.it (A.B.)
[2] Department of Precision Medicine, University of Campania Luigi Vanvitelli, Via L. De Crecchio 7, 80138 Naples, Italy; lucia.capasso2@unicampania.it (L.C.); angela.nebbioso@unicampania.it (A.N.); paola.bontempo@unicampania.it (P.B.)
[3] Department of Pharmacy, University of Naples Federico II, Via Montesano 49, 80131 Naples, Italy; mariano.stornaiuolo@unina.it (M.S.); carmina.sirignano@unina.it (C.S.)
[4] Department of Pharmacy, University of Salerno, via Giovanni Paolo II 132, 84084 Fisciano (Salerno), Italy; piacente@unisa.it (S.P.); acerulli@unisa.it (A.C.)
[5] Agronomist consultant, Via S. Moccia 2/B, 83100 Avellino, Italy; nadia.marallo@libero.it
[6] National Research Council (CNR), Institute of Food Science (ISA), Via Roma 64, 83100 Avellino, Italy; deborah.giordano@isa.cnr.it (D.G.); angelo.facchiano@isa.cnr.it (A.F.)
[7] National Research Council (CNR), Institute of Biosciences and BioResources (IBBR), Via Università 133, 80055 Portici (Naples), Italy
* Correspondence: drigano@unina.it (D.R.); luigi.demasi@ibbr.cnr.it (L.D.M.)
† These authors contributed equally to this work.

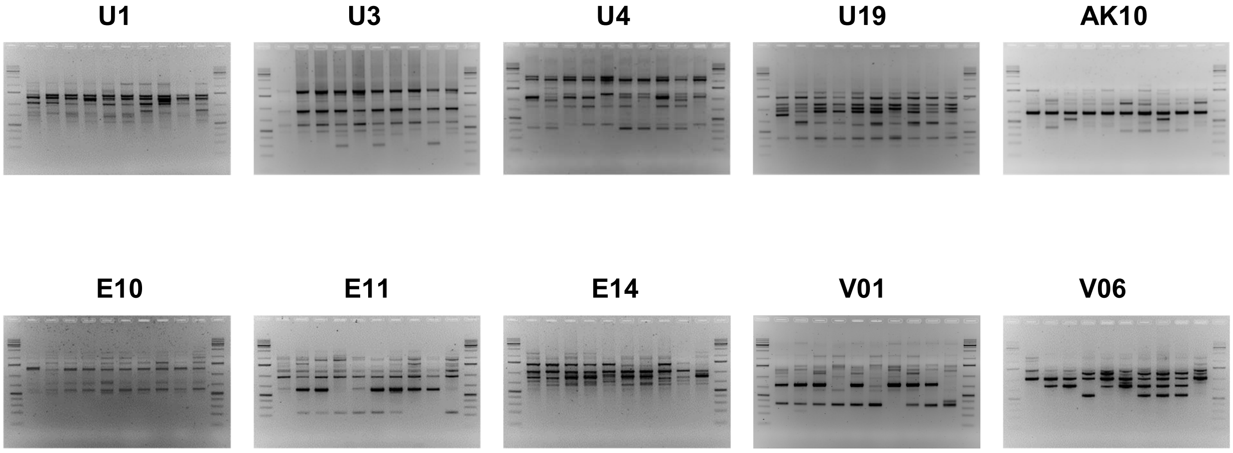

Figure S1. **Genetic diversity of purple carrot 'Purple Sun' detected by RAPD molecular analysis**. Comparison of the DNA profiles obtained after separation by agarose gel electrophoresis in 10 individual plants for each panel using the indicated RAPD arbitrary primer. First and last lane of each panel: GeneRuler 1 kb Plus DNA Ladder (Thermo Fisher Scientific) as molecular weight marker, containing three darkest bands consisting of 5000, 1500, and 500 bp. Central lanes of each panel: 10 individual plants of purple carrot (CAR1-CAR10).

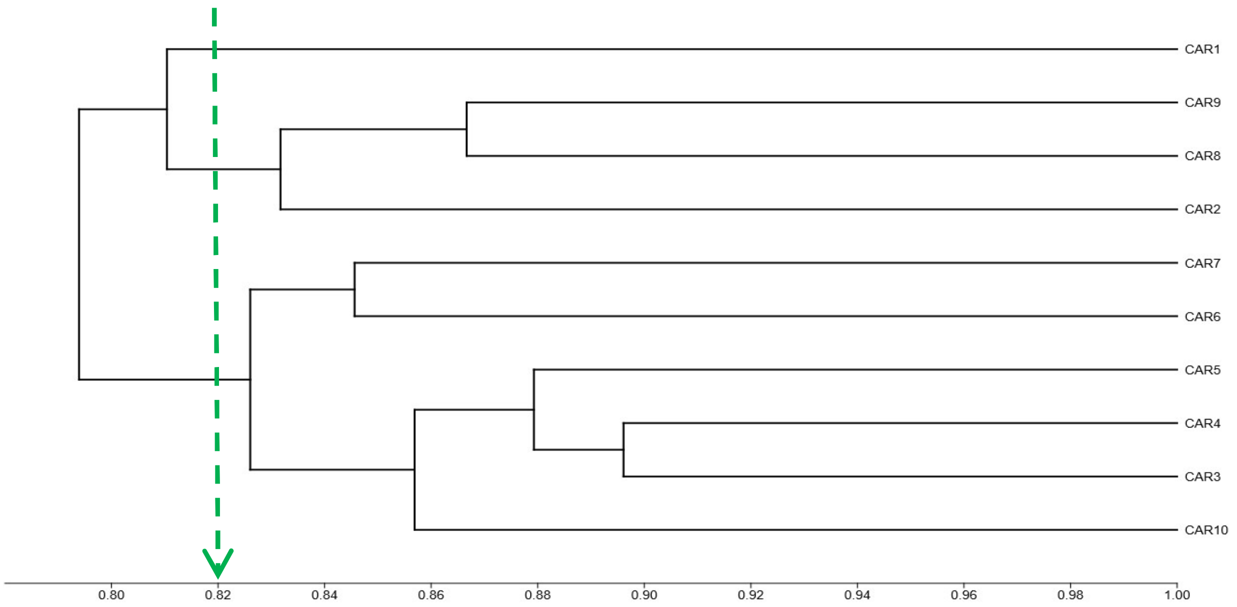

Figure S2. **Dendrogram based on UPGMA clustering of RAPD markers from purple carrot 'Purple Sun' plants**. Genetic data were from 10 individual plants (CAR1-10) belonging to the population of the purple carrot 'Purple Sun' under investigation. Numerical scale indicates the Dice's genetic similarity coefficient (Dc). The average Dc of 0.82 is used as cut-off line (green arrow) to detect the main clusters.

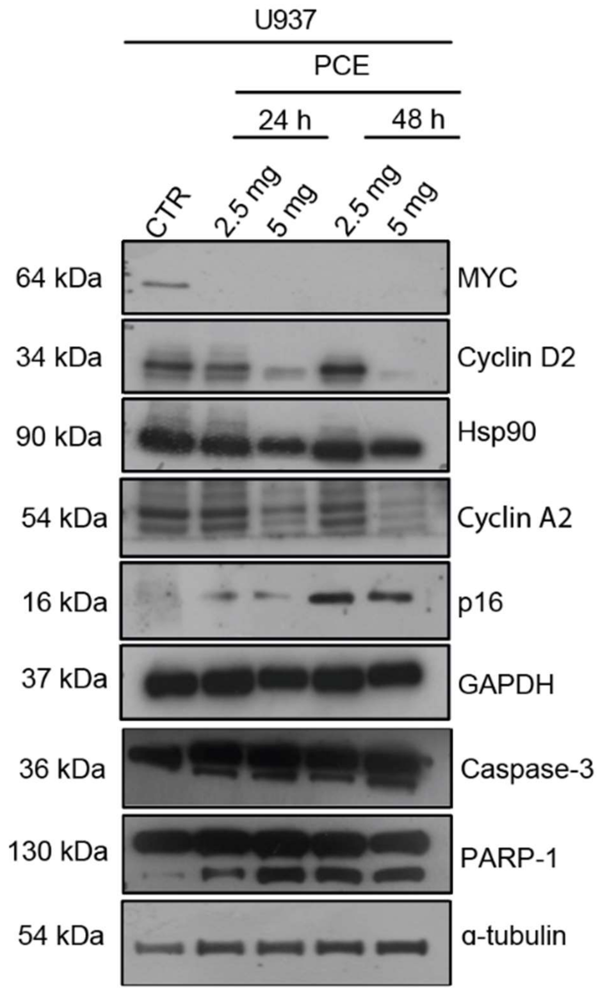

Figure S3. **Purple carrot extract (PCE) modulated the expression of important cell cycle players in hematological cancer cells**. Western blot of the indicated proteins after 2.5 and 5.0 mg/mL PCE treatment at 24 and 24 hours on U937 cells, as compared to untreated cells (CTR). Hsp90, GAPDH, and α-tubulin were used as respective loading controls.

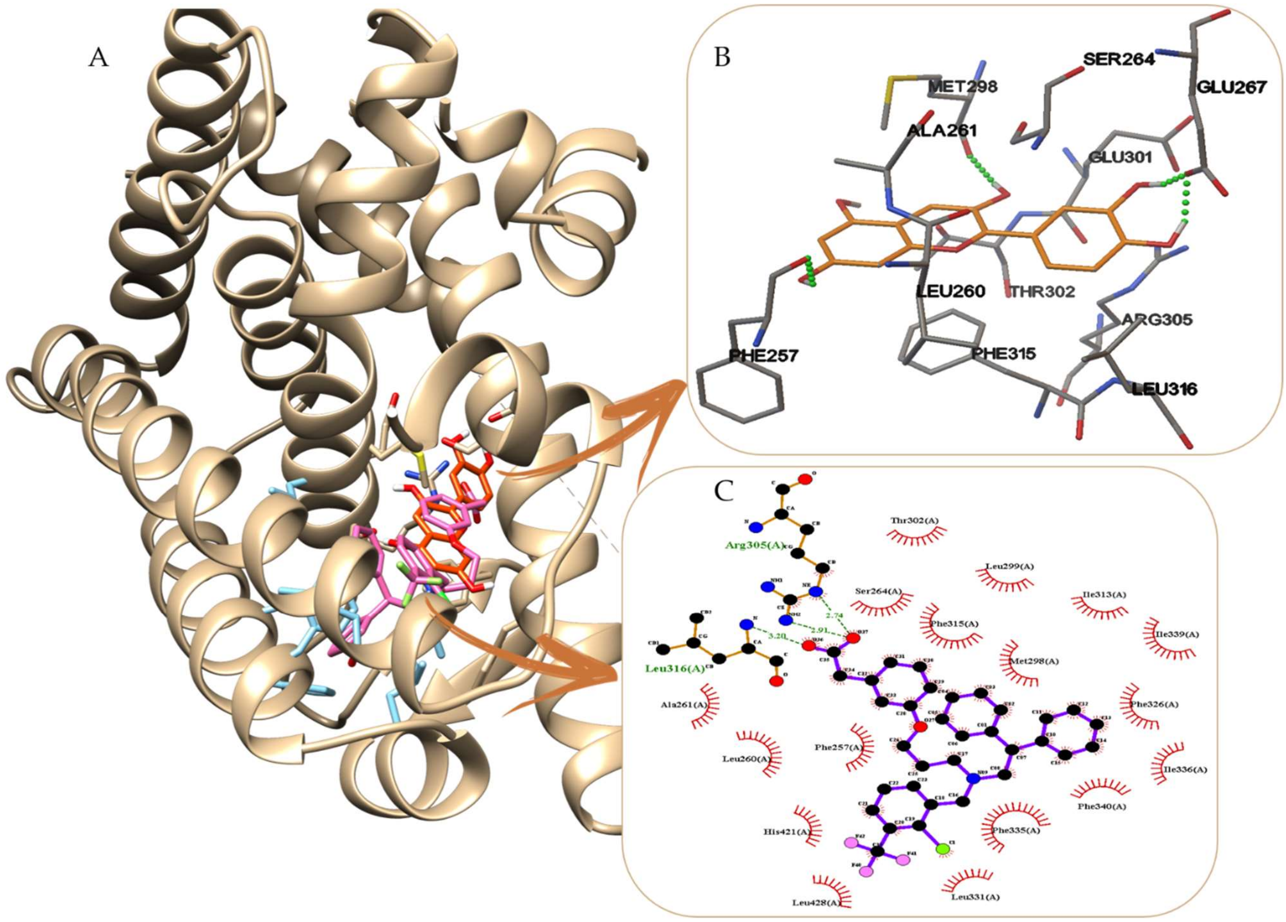

Figure S4. Interactions of cyanidin and GW3965 in the agonist pocket of LXR-alpha.

Panel A: overlap of the cyanidin (in orange sticks) and the GW3965 agonist (in pink sticks) in the agonist pocket of the LXR-alpha protein represented in beige cartoons. Image created with Chimera 1.14.

Panel B: details of the interaction, obtained by focused docking simulation, among cyanidin (orange sticks) and the residues of LXR-alpha, represented by green dots the H-bonds. Image created with AutodockTools.

Panel C: detail of the interaction detected by the focused docking results about the GW3965 (violet lines), green labels and lines indicated residues involved in the H-bonds formation. Image created with LigPlot.

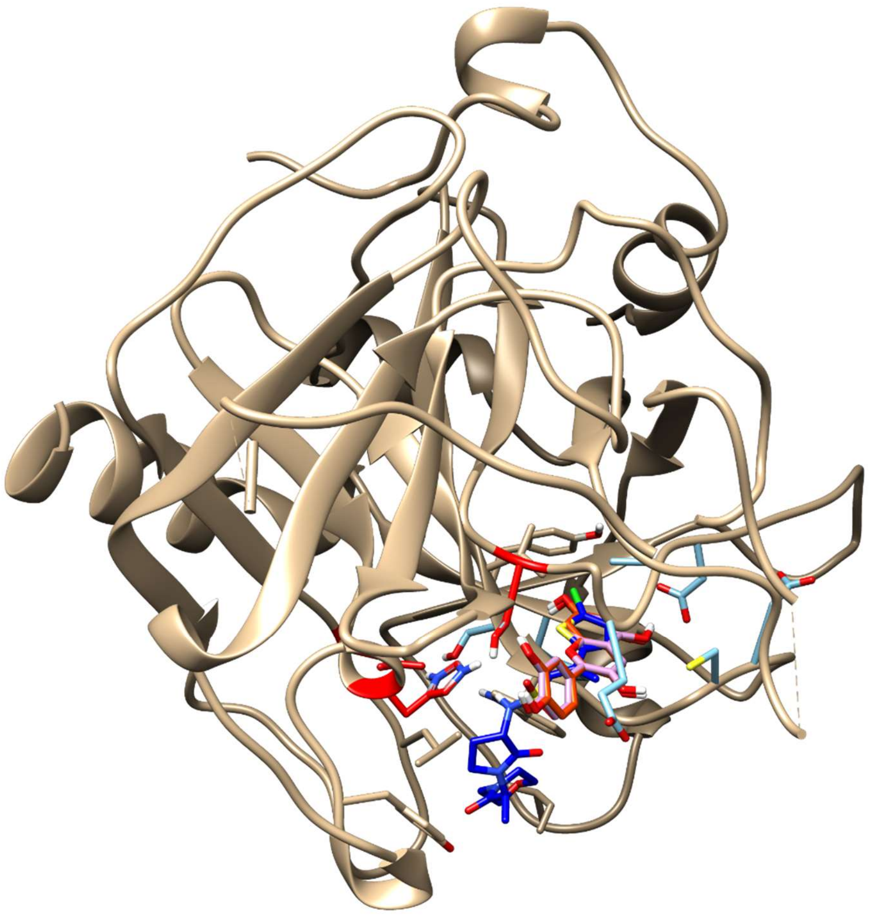

Figure S5. Interactions of cyanidin and SI in the active site pocket of thrombin. Overlap of the cyanidin binding poses, obtained by focused (orange sticks) and blind docking (pink sticks), with the SI inhibitor (blue sticks). The catalytic residues are highlighted in red sticks, while the other interacting residues are highlighted in cyan sticks. Cyanidin being smaller is superimposable only to the first part of SI (blue sticks), despite that is still able to interact with the catalytic residue S195. Image created with Chimera 1.4.

Table S1. Details of the 10-mer arbitrary primers used for DNA analysis of the purple carrot ‘Purple Sun’ in study.

N.	Primer name	Primer sequence (5’ to 3’)	G+C (%)
1	AK10	CAAGCGTCAC	60
2	E10	CACCAGGTGA	60
3	E11	GAGTCTCAGG	60
4	E14	TGCGGCTGAG	70
5	U1	AGGGGTCTTG	60
6	U3	GGGTTTAGGG	60
7	U4	GACAGACAGG	60
8	U19	TGGGAACGGT	60
9	V01	TGACGCATGG	60
10	V06	ACGCCCAGGT	70

Table S2. Similarity/distance matrix* based on Dice coefficient between pairs of individual samples** in study belonging to the purple carrot 'Purple Sun'.

Sample	**CAR10**	**CAR9**	**CAR8**	**CAR7**	**CAR6**	**CAR5**	**CAR4**	**CAR3**	**CAR2**	**CAR1**
**CAR10**		0.29	0.25	0.18	0.16	0.16	0.13	0.13	0.24	0.21
**CAR9**	0.71		0.13	0.23	0.21	0.21	0.23	0.22	0.18	0.17
**CAR8**	0.75	0.87		0.16	0.16	0.16	0.17	0.13	0.16	0.19
**CAR7**	0.82	0.77	0.84		0.15	0.20	0.19	0.18	0.21	0.25
**CAR6**	0.84	0.79	0.84	0.85		0.18	0.16	0.15	0.22	0.27
**CAR5**	0.84	0.79	0.84	0.80	0.82		0.13	0.11	0.16	0.17
**CAR4**	0.87	0.77	0.83	0.81	0.84	0.87		0.10	0.24	0.21
**CAR3**	0.87	0.78	0.87	0.82	0.85	0.89	0.90		0.17	0.18
**CAR2**	0.76	0.82	0.84	0.79	0.78	0.84	0.76	0.83		0.20
**CAR1**	0.77	0.83	0.81	0.75	0.73	0.83	0.79	0.82	0.80	

* At the bottom left, Dice's genetic symilarity coefficient (Dc); at the top right, genetic distance coefficient complementary to one of Dc.
** CAR1-10: 10 individual samples of purple carrot 'Purple Sun'.

Table S3. **Results of the molecular docking simulations**. In the pocket of interaction, column residues underlined are involved in H-bond with the ligand, while the ones highlighted in red are active site residues. The number of poses in cluster indicates the number of different conformations detected for the ligand in that specific pocket, calculated on a sampling of 100 total poses. On the lowest side of the table, the interaction areas detectable from the crystal structures are reported as further control of the re-docking procedures and of consequence of the docking simulations.

**Protein**	**Ligand**	**Docking type**	**Lowest Binding Energy (kcal/mol)**	**Mean Binding Energy (kcal/mol)**	**No. of poses in cluster**	**Pocket of Interaction**
SIRT6 (control)	Cyanidin	BLIND	-8.39	-7.71	20	R126-L129-E131-G134-E140-Q147-L159-T184-L186-P193-D196
			-7.31	-6.75	6	A53-S56-I61-P62-F64-V70-F82-F86-N114-V115-D116-M136-M157
		FOCUSED	-7.31	-6.73	51	A53-I61-P62-F64-V70-F82-F86-N114-V115-D116-M136-M157
LXR-alpha	Cyanidin	BLIND	-8.58	-7.72	24	F257-L260-A261-S264-E267-M298-E301-T302-R305-F315-L316
	GW3965 (control)	BLIND	-7.67	-7.18	2	Q221-S303-R304-R305-Y306-N307-M346-N347-Q350-L351-D353-F356
	Cyanidin	FOCUSED	-8.69	-7.95	76	F257-L260-A261-S264-E267-M298-E301-T302-R305-F315-L316
	GW3965 (control)	FOCUSED	-14.38	-12.65	38	F257-L260-A261-S264-M298-L299-T302- R305- I313-F315-L316-F326-L331-F335-I336-I339-F340-H421-L428
Thrombin	Cyanidin	BLIND	-7.66	-7.23	55	D189-A190-C191-E192-G193-S195-V213-S214-W215-G216-G219-C220-G226-F227-Y228
	SI (control)	BLIND	-9.70	-8.76	26	H57-Y60A-N98-L99-D102- I174-D189-A190-S195-V213-S214-W215-G216
		BLIND	-8.62	-8.01	9	E97-N98-L99-I174-D189-A190-C191-E192-V213-W215-G216-G219-C220-G226
	Cyanidin	FOCUSED	-7.71	-7.28	58	D189-A190-C191-E192-G193-S195-V213-S214-W215-G216-G219-C220-G226-F227-Y228
	SI (control)	FOCUSED	-10.41	-9.06	19	H57-Y60A-W60D-E97A-N98-L99-D102-I174-D189-A190-C191-E192-S195-V213-S214-W215-G216-G226-Y228

Crystallographic references

**PDB code**	**Protein**	**Ligand**	**Pocket of interaction**
6QCH	SIRT6	Cyanidin	V115-D116-I61-P62-F64-V70-P80-F82-F86-M136-M157
3IPQ	LXR-alpha	Gw3965	F257-L260-S264-E267-I295-M298-L299-T302-I313-R305-F315-L316-F326-F335-I336-I339-F340-H421-W443
2JH0	Thrombin	SI	H57-Y60A-W60D-D189-A190-C191-E192-V213-W215-G216-G219-C220-G226
